# Solid Phase Formylation of N-Terminus Peptides

**DOI:** 10.3390/molecules21060736

**Published:** 2016-06-04

**Authors:** Anna Lucia Tornesello, Marina Sanseverino, Franco Maria Buonaguro

**Affiliations:** 1 CROM, Istituto Nazionale Tumori “Fondazione G. Pascale”—IRCCS, 80131 Napoli, Italy; 2 Inbios Srl, via Pietro Castellino 111, 80131 Napoli, Italy; info@inbios.it; 3 Molecular Biology and Viral Oncology Unit, Research Department, Istituto Nazionale Tumori “Fondazione G.Pascale”—IRCCS, 80131 Napoli, Italy; fm.buonaguro@istitutotumori.na.it

**Keywords:** formylation, peptide, solid phase synthesis, amines

## Abstract

Formylation of amino groups is a critical reaction involved in several biological processes including post-translational modification of histones. The addition of a formyl group (CHO) to the N-terminal end of a peptide chain generates biologically active molecules. N-formyl-peptides can be produced by different methods. We performed the N-formylation of two chemotactic hexapetides, Met1-Leu2-Lys3-Leu4-Ile5-Val6 and Met1-Met2-Tyr3-Ala4-Leu5-Phe6, carrying out the reaction directly on peptidyl-resin following pre-activation of formic acid with *N*,*N*-dicyclohexylcarbodiimmide (DCC) in liquid phase. The overnight incubation at 4 °C resulted in a significant increase in production yields of formylated peptides compared to the reaction performed at room temperature. The method is consistently effective, rapid, and inexpensive. Moreover, the synthetic strategy can be applied for the formylation of all primary amines at N-terminus of peptide chains or amino groups of lysine side-chains in solid phase.

## 1. Introduction

Protein post-translational modifications by the covalent addition of functional groups to N-terminal amino acids greatly expand their biological properties in prokaryotic as well eukaryotic organisms. The most common reactions include acetylation, methylation, pyroglutammate formation, myristoylation, amidation, glycosyl phosphatidylinositol (GPI) attachment, and formylation [1]. N-formyl peptides derived from cleavage of bacterial and mitochondrial proteins have an important role in host defense against microbial agents for their ability to chemo attract phagocytic leukocytes to the site of infection or tissue damage [2]. Moreover, formylation is one of the many post-translational modifications occurring in histone protein which modulates chromatin conformation and gene activation [3]. In prokaryotic as well as eukaryotic cells, an increasing number of modifying enzymes have shown to contain formylation domains [4].

In organic synthesis, several methods for the formylation of amines have been developed mainly based on the dissolution of formylating reagents in liquid phase [5]. The main amine formylating reagents include chloroform [6], formic acid and derivatives [7], paraformaldehyde [8], methanol [9], carbon dioxide [10], and carbon monoxide [11]. Nevertheless, formic acid being inexpensive and easily available represents the preferential reagent to produce formylated molecules in high yields. The use of solid-supported reagents and microwave irradiation allows for the automation and acceleration the organic syntheses [12]. Recently, several chemical techniques have been published to convert amines into the corresponding N-formamides in good yields [5,6,7,8,9,10,11,12,13,14,15,16,17]. Waki and Meienhofer developed an efficient procedure for the preparation of Nα-formyl amino acid *tert*-butyl esters with minimal racemization to be utilized as starting material for peptide synthesis. The method is based on the mixing of formic acid and *N*,*N*-dicyclohexylcarbodiimmide (DCC) in the presence of chloroform to form the active formylating reagent, which was added to solutions of *tert*-butyl amino acid esters [17]. However, a major issue in the reported chemical technique of formylation remains the removal of side products (*i.e.*, urea) from synthesized molecules with lengthy purifications and reduction of the reaction yields.

We developed a simple method for the N-formylation of primary amines in solid phase synthesis, which affords high quantities of N-formylated peptides and to easily remove the side products by resin washings.

## 2. Results and Discussion

**Scheme 1:**
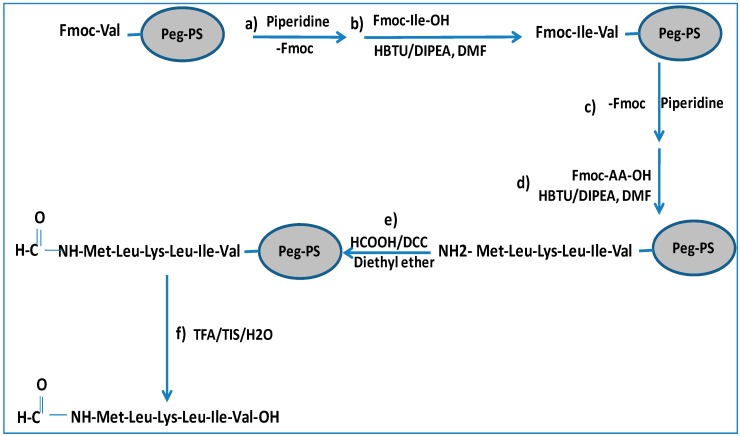
Synthesis of N-formylated peptide: (**a**) Fmoc deprotection of preloaded resin with the first amino acid (20% piperidine in *N*,*N*-dimethylformamide (DMF) for 10 min); (**b**) amino acid coupling (HBTU/Fmoc-AA-OH/DIPEA (4:4:8), reaction time 20 min); (**c**) Fmoc deprotection (as for step a); (**d**) amino acid coupling as for step (**b**) for all amino acids; (**e**) formylation of the peptidyl-resin by overnight incubation at 4 °C with a formylating reagent produced by incubation of formic acid and *N*,*N*-dicyclohexylcarbodiimmide (DCC) in diethyl ether at 0 °C for 4 h; (**f**) Cleavage of formylated peptides from the resin by 3 h incubation with a solution of TFA/TIS/H_2_O (95:5:5). See the Abbreviations section for definitions.

**Figure 1:**
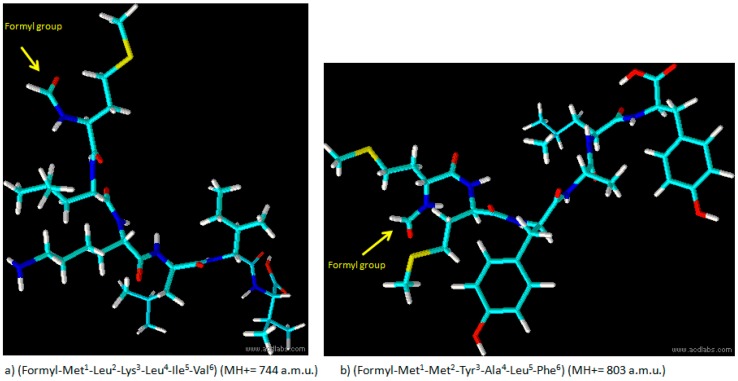
Structure of formylated peptide (**a**) and formylated peptide (**b**).

We synthesized two peptides (peptide “a” and peptide “b”) corresponding to subunit 4 and 6 of human mitochondrial NADH dehydrogenase, respectively. The formylated sequences act as chemoattractants for leukocytes, can trigger a dramatic increase in the phosphorylation levels of ERK1/2, and are able to change the cytosolic calcium concentration in promyelocytic HL-60 cell line, stably expressing either FPR1 or FPR2 [18]. The synthesis of peptide “a” is illustrated in Scheme 1. The protocol is based on the addition of a formylation group to the N-terminus of a peptidyl-resin followed by cleavage to obtain the final formylated peptides (Met1-Leu2-Lys3-Leu4-Ile5-Val6 (MH+ = 744 a.m.u.) and Met1-Met2-Tyr3-Ala4-Leu5-Phe6 (MH+ = 803 a.m.u.)) illustrated in Figure 1.

**Figure 2:**
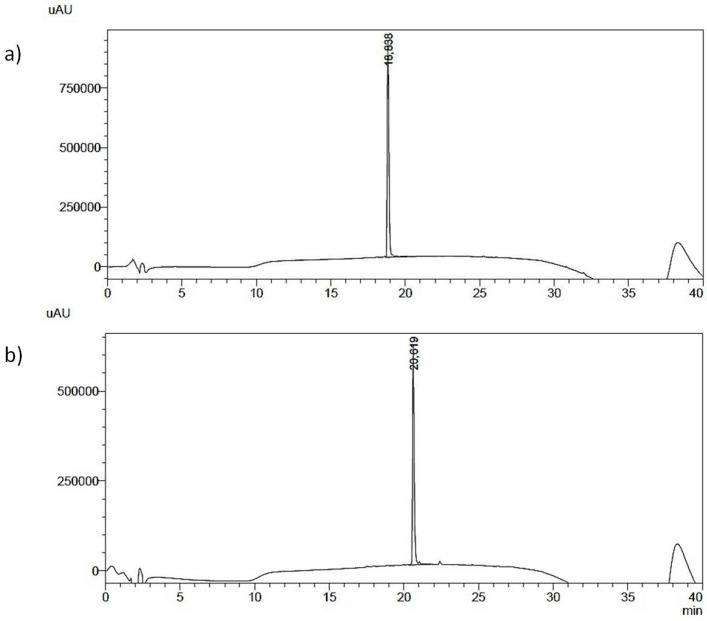
HPLC elution profile of purified peptides (**a**,**b**).

**Figure 3:**
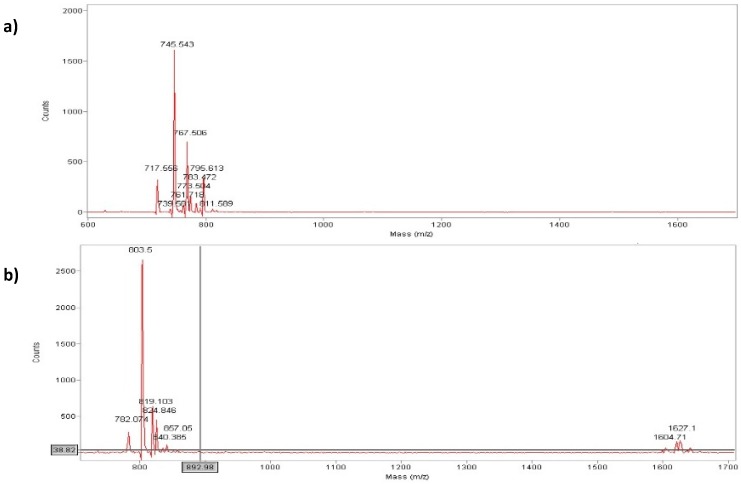
Maldi-Tof analysis of peptide (**a**) (MH+ = 744 a.m.u) and peptide (**b**) (MH+ = 803 a.m.u.).

The formylated peptides were synthesized using a standard Fmoc-based solid phase peptide synthesis [19,20]. In particular, the preloaded Fmoc-Val-PEG-PS resin was employed for peptide “a” and Fmoc-Phe-PEG-PS was employed for peptide “b”. The formylation reaction was performed after the complete assembly of the peptide chains on solid support by incubation with the formylating reagent obtained, in liquid phase, by mixing formic acid with DCC in diethyl ether at 0 °C. The formylating solution, filtered to remove the side product urea and concentrated by rotary evaporator, was added to the peptidyl-resins with DIPEA (diisopropylethylamine) in DMF (*N*,*N*-dimethylformamide). The reaction was carried out at 4 °C overnight. Low temperatures were important for preventing formic acid decomposition. The outcome of the N-formylation reaction was verified by the Kaiser test. The final step consisted in the cleavage of the formylated peptide from the solid support with an aqueous acidic solution containing TFA (trifluoroacetic acid) and the addition of TIS (triisopropylsilane) as scavengers to prevent by-product formation from electrophilic intermediates present in the cleavage process. The final products were obtained in good yield (70%–75%) and in high purity after RP-HPLC purification. The analyses by HPLC and MALDI-Tof of purified products are reported in Figure 2 and Figure 3, respectively.

Different temperatures were used in preliminary reactions in order to identify optimal conditions. We found that the temperature has a significant effect on the formylating reaction efficiency. Reactions carried out between 10 °C and room temperature were much faster but provided very low yields (5% of purified product) in comparison to the reactions performed at 4 °C (yield 70%–75%). The best yields were obtained by incubating formic acid with DCC at 0 °C to form the active formylating reagent and at 4 °C to perform the formylation of N-terminus peptides. As reported in the literature, good yields of formylation (50%–90%) have been also obtained in liquid phase [5]. However, the synthesis in liquid phase is more laborious, requiring a higher number of steps. Liquid phase reactions have several disadvantages such as expensive reagents, the formation of side products, thermal instability, and difficult accessibility to reagents. Chemical modifications in solid phase facilitate the entire procedure by the elimination of tedious purification steps that often contribute to lower the yield. Excess of reagents are removed by filtration.

The method that we have developed is applicable in solid phase for the formylation of all primary amines at N-terminus of peptide chains and for the formylation of amino groups of lysine side-chains after the selective removing of side-chain protecting groups on resins.

## 3. Materials and Methods

All reagent were obtained from commercial suppliers and were used without further purification. Preloaded resins were purchased from Rapp Polymere (Tuebingen, Germany), and protected amino acids were purchased from AGTC Bioproducts (East Riding of Yorkshire, UK). Formic acid and all other chemicals was provided by Sigma-Aldrich Srl (Milano, Italy). The analytical and preparative reverse phase HPLC (RP-HPLC) columns were purchased from Phenomenex (Castelmaggiore, Italy). Maldi-Tof spectral analyses were carried out on MALDI-Tof Voyager-DE mass spectrometer by Perspective Biosystems (Framingham, MA, USA).

### 3.1. Peptides Synthesis

Peptides Met1-Leu2-Lys3-Leu4-Ile5-Val6 (a) and Met1-Met2-Tyr3-Ala4-Leu5-Phe6 (b) were synthesized through solid phase strategy in continuous flow with automatic synthesizer Syro by Multisynthec using the Fmoc (9-fluorenylmethoxycarbonyl) chemistry. The supports Fmoc-Val-PEG-PS (resin substitution: 0.23 mmol/g, from Applied Biosystem, (Foster City, California, CA, USA) and Fmoc-Phe-PEG-PS (resin substitution: 0.20 mmol/g, from Applied Biosystem) allow for the obtainment of the peptides as acid at C-terminal. The synthesis was made on a 0.05-mmol scale for each peptide, using as a protected amino acid Fmoc-Met-OH, Fmoc-Leu-OH, Fmoc-Lys(Boc)-OH, Fmoc-Ile-OH, Fmoc-Val-OH, Fmoc-Tyr(t-Bu)-OH, Fmoc-Ala-OH, and Fmoc-Phe-OH. The automatic synthesis proceeded through Fmoc deprotection steps [piperidine:DMF, 0.2:1 (*v*/*v*)] and Fmoc-aminoacid coupling steps [Support:Fmoc-aminoacid:HBTU:DIEA, 1:4:4:8].

### 3.2. Formylation and Cleavage from the Solid Support

The formylating reagent for each peptide was produced by incubation in a flask of formic acid (0.94 mL, 25 mmol) and DCC (2.57 g, 12.5 mmol) with diethyl ether (14.5 mL) at 0 °C for 4 h. This reagent was then filtered to remove DCU (dicyclohexylurea), and the total volume (about 16 mL) was reduced to 2 mL by evaporation under vacuum. The final solution was added to each peptidyl-resin in DMF (1 mL) with DIEA (20 microliters, 125 mmol), and the reaction tube was kept at 4 °C overnight to avoid formic acid decomposition. Completeness of formyl coupling reactions was monitored by the ninydrin test of Kaiser. The resin was washed consecutively with DMF (2 times), with dry DCM (dichloromethane) (3 times) and dried *in vacuo*. Each linear peptide was cleaved from the solid support, and amino acid side-chains were simultaneously deprotected by suspending the resin in 2 mL of a mixture of TFA:water:TIS [95:2.5:2.5 (*v*/*v*)] for three hours.

The resin was then removed by filtration under a reduced pressure, and the filtrate poured into eight volumes of cold ether to achieve a good peptide precipitation. The suspensions were centrifuged, and the ether carefully decanted. After a further ether wash, the peptides were dissolved in 0.1% TFA (*v*/*v*) in water (3 mL) and then lyophilized.

### 3.3. Peptides Analysis and Purification

Analytical RP-HPLC runs were carried out on a Shimadzu LC-10 ADVP (detector: SPDM) apparatus using a C12 column by Phenomenex (column 4.6 × 150 mm; eluent A: 0.1%TFA in water; eluent B: 0.1%TFA in acetonitrile; gradient: from 5%B to 65%B in 20 min; flow 1.0 mL·min^−1^). Preparative RP-HPLC was carried out on a Shimadzu LC-8 apparatus equipped with an UV Shimadzu detector SPD-10AVP using a Phenomenex C12 column, 22 × 250 mm with a flow rate of 20 mL·min^−1^ and with a linear gradient from 5%B to 65%B in 30 min. The main peaks of the analytical chromatogram, with Rt = 18.8 min for peptide “a” and with Rt = 20.6 min for peptide “b” were confirmed by MALDI-TOF spectrometry (peptide “a” (MH+ = 744 a.m.u.) and peptide “b” (MH+ = 803 a.m.u.).

## 4. Conclusions

We developed a simple and efficient method for N-formylation of peptides using the standard Fmoc-based solid phase peptide synthesis. This method affords good yields and high purity products in comparison with the liquid phase synthesis, which render the separation of the byproduct difficult, and the formylation in acidic condition, which might cause some premature removal of the peptide from the resin. The formylation reaction performed directly on solid phase incubating the peptidyl-resin with the formylating reagent, obtained by reaction, in liquid phase, of formic acid with DCC in diethyl ether, is especially recommended because the reagents are easy to handle, inexpensive, commercially available, and upscalable for industrial processes.

## Abbreviations

The following abbreviations are used in this manuscript:

DCC	*N*,*N*-dicyclohexylcarbodiimmide
DCM	Dichloromethane
DIPEA	diisopropylethylamine;
DMF	*N*,*N*-dimethylformamide
Fmoc	9-fluorenylmethoxycarbonyl
HBTU	2-(1*H*-benzotriazol-1-yl)-1,1,3,3-tetramethyluronium hexafluorophosphate
TFA	trifluoroaceticacid
TIS	triisopropylsilane
